# *NELFCD* and *CTSZ* loci are associated with jaundice-stage progression in primary biliary cholangitis in the Japanese population

**DOI:** 10.1038/s41598-018-26369-6

**Published:** 2018-05-23

**Authors:** Nao Nishida, Yoshihiro Aiba, Yuki Hitomi, Minae Kawashima, Kaname Kojima, Yosuke Kawai, Kazuko Ueno, Hitomi Nakamura, Noriyo Yamashiki, Tomohiro Tanaka, Sumito Tamura, Akira Mori, Shintaro Yagi, Yuji Soejima, Tomoharu Yoshizumi, Mitsuhisa Takatsuki, Atsushi Tanaka, Kenichi Harada, Shinji Shimoda, Atsumasa Komori, Susumu Eguchi, Yoshihiko Maehara, Shinji Uemoto, Norihiro Kokudo, Masao Nagasaki, Katsushi Tokunaga, Minoru Nakamura

**Affiliations:** 1 Department of Human Genetics, Graduate School of Medicine, The University of Tokyo, Tokyo, Japan; 2 Genome Medical Science Project, National Center for Global Health and Medicine, Ichikawa, Japan; 3 Clinical Research Center, National Hospital Organization (NHO) Nagasaki Medical Center, Omura, Japan; 4 Division of Biomedical Information Analysis, Department of Integrative Genomics, Tohoku Medical Megabank Organization, Tohoku University, Sendai, Japan; 5 Organ Transplantation Service, The University of Tokyo, Tokyo, Japan; 6 Hepatobiliarypancreatic Surgery Division, Artificial Organ and Transplantation Division, Department of Surgery, Graduate School of Medicine, The University of Tokyo, Tokyo, Japan; 7 Division of Hepato-Biliary-Pancreatic and Transplant Surgery, Department of Surgery, Graduate School of Medicine, Kyoto University, Kyoto, Japan; 8 Department of Surgery and Science, Kyushu University Graduate School of Medical Sciences, Fukuoka, Japan; 9 Department of Transplantation and Digestive Surgery, Nagasaki University Graduate School of Biomedical Sciences, Nagasaki, Japan; 10 Department of Medicine, Teikyo University School of Medicine, Tokyo, Japan; 11 Department of Human Pathology, Kanazawa University Graduate School of Medicine, Kanazawa, Japan; 12 Department of Medicine and Biosystemic Science, Kyushu University Graduate School of Medical Sciences, Fukuoka, Japan; 13 Department of Hepatology, Nagasaki University Graduate School of Biomedical Sciences, Omura, Japan; 14 Headquarters of PBC-GWAS Consortium in Japan (PBCJPN), National Hospital Organization (NHO) Study Group for Liver Disease in Japan (NHOSLJ), and gp210 Working Group in Intractable Liver Disease Research Project Team of the Ministry of Health and Welfare in Japan, Omura, Japan

## Abstract

Approximately 10–20% of patients with primary biliary cholangitis (PBC) progress to jaundice stage regardless of treatment with ursodeoxycholic acid and bezafibrate. In this study, we performed a GWAS and a replication study to identify genetic variants associated with jaundice-stage progression in PBC using a total of 1,375 patients (1,202 early-stage and 173 jaundice-stage) in a Japanese population. SNP rs13720, which is located in the 3′UTR of *cathepsin Z* (*CTSZ*), showed the strongest association (odds ratio [OR] = 2.15, *P* = 7.62 × 10^−7^) with progression to jaundice stage in GWAS. High-density association mapping at the *CTSZ* and *negative elongation factor complex member C/D* (*NELFCD*) loci, which are located within a strong linkage disequilibrium (LD) block, revealed that an intronic SNP of *CTSZ*, rs163800, was significantly associated with jaundice-stage progression (OR = 2.16, *P* = 8.57 × 10^−8^). In addition, eQTL analysis and *in silico* functional analysis indicated that genotypes of rs163800 or variants in strong LD with rs163800 influence expression levels of both *NELFCD* and *CTSZ* mRNA. The present novel findings will contribute to dissect the mechanism of PBC progression and also to facilitate the development of therapies for PBC patients who are resistant to current therapies.

## Introduction

Primary biliary cholangitis (PBC, MIM 109720), previously called primary biliary cirrhosis, is a chronic autoimmune liver disease characterized by the destruction of intrahepatic bile ducts and progressive cholestasis, leading to liver cirrhosis, jaundice, and hepatic failure^1^. Although treatment with ursodeoxycholic acid (UDCA), either as monotherapy or in combination with bezafibrate, is very effective for normalization of liver functions and prevention of PBC progression^2,3^, approximately 10–20% of PBC patients are resistant to these treatments and ultimately progress to hepatic failure^4–6^. However, the mechanisms underlying the progression of PBC are poorly understood, and the existence of two different types of PBC progression, jaundice type and portal hypertension type, has been proposed^4^.

The high concordance rate of PBC in monozygotic twins in comparison with dizygotic twins, along with the familial clustering of PBC patients, indicates that strong genetic factors are involved in the development of PBC^7^. Indeed, genome-wide association studies (GWASs) and subsequent meta-analyses have identified susceptibility loci for PBC, including *Human Leukocyte Antigen* (*HLA*) and more than 30 non-*HLA* loci, in individuals of European descent^8–15^. In addition, GWASs in the Japanese population identified three novel PBC susceptibility loci, including *tumor necrosis factor superfamily member 15* (*TNFSF15*), *POU domain class 2 associating factor 1* (*POU2AF1*), and *protein kinase C beta* (*PRKCB*), which have not been identified in European descent^16,17^. The *IL21*, *IL4R/IL21R*, *CD28/CTLA4/ICOS*, *CD58*, *ARID3A*, *IL16*, and *CSNK2A2/CCDC113* loci were also identified as novel susceptibility loci for PBC in Han Chinese subjects^18^. These GWASs indicate that similar autoimmune pathways of dendritic cell, T-cell, and B-cell activation and/or differentiation, including MAPK-, phosphatidylinositol-, TNF superfamily-, and NFκB-signaling, contribute to the development of PBC in all populations, although the specific PBC susceptibility genes differ somewhat among Europeans and East Asians.

Several genetic loci, including *MDR3*, *ITGAV*, *CTLA4*, *CYP7A1*, *HNF4A*, and *PPARGC1A*, have been reported to be associated with progression to jaundice stage and/or hepatic failure in PBC^19–22^. However, these results were obtained by candidate gene approaches that were insufficiently robust. Previously, genetic variants associated with progression in PBC had not yet been identified by whole-genome approaches. Therefore, we performed a GWAS to identify genetic variants associated with jaundice-stage progression in a Japanese population.

## Results

### GWAS to identify genetic factors associated with disease progression in PBC

Of 1,375 Japanese PBC patients enrolled in this study, 173 were diagnosed as late stage with jaundice (T.bil ≥ 2 mg/dL) regardless of treatment with UDCA or UDCA plus bezafibrate, whereas the remaining 1,202 patients were diagnosed as early stage at the end of observation (Table 1). For the GWAS, we genotyped 1,125 samples (including 150 samples from jaundice-stage patients and 975 from early-stage patients) using the Affymetrix Axiom Genome-Wide ASI 1 Array. No samples were excluded by Dish QC (threshold ≥0.82 for each sample). Genotype calls of 1,125 samples for approximately 600,000 SNPs were determined using the Genotype Console v4.1 software; no samples were excluded based on overall call rate (threshold >0.97). Among the 1,125 samples, no related samples were identified in identity-by-descent testing. Moreover, principal component analysis using 1,125 samples and HapMap samples (43 JPT, 40 CHB, 91 YRI, and 91 CEU samples) showed a clear cluster of studied samples overlapping only with HapMap JPT (Supplementary Figure 1); ultimately, the average overall call rates of 150 jaundice-stage PBC patients and 975 early-stage PBC patients were 99.37% (97.47–99.80) and 99.41% (97.32–99.81), respectively.

**Table 1:** Demographics and Clinical data of 1,375 Japanese PBC patients.

Characteristics	Jaundice-stage (n = 173)	Early-stage (n = 1,202)	*P*
Male/female	20/153	149/1,053	n.s.
Age (years): median (range)	51 (27–76)	60 (23–89)	<0.001
AMA positivity (%)	77.9	84.1	n.s.
Anti-gp210 antibody positivity (%)	58.7	20.4	<0.001
Anti-centromere antibody positivity (%)	15.1	23.6	<0.05

n.s.: not significant, AMA: anti-mitochondrial antibody.

For SNP quality control, we applied the following thresholds during data cleaning (Supplementary Table 1): Hardy-Weinberg equilibrium *P* > 0.001, SNP call rate >95%, and MAF >5% in both early- and jaundice-stage PBC patients. All cluster plots for SNPs with *P* < 0.0001 from a chi-square test of the allelic model were checked by visual inspection, and SNPs with ambiguous genotype calls were excluded. Of the SNPs on autosomal chromosomes, a total of 426,245 SNPs passed the quality control filters and were used for the association analysis.

**Figure 1:**
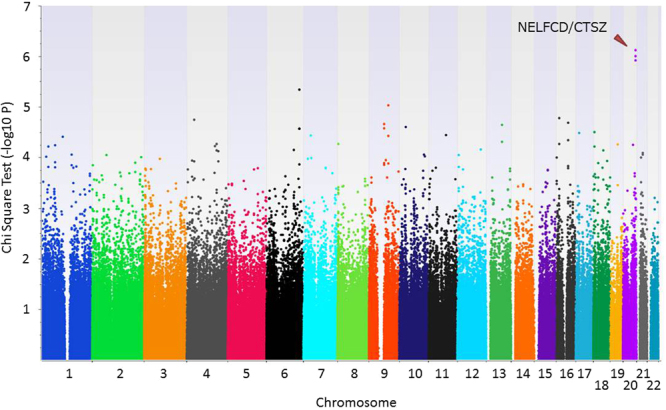
Genome-wide association results. From 1,125 Japanese samples, including 150 jaundice-stage and 975 early-stage PBC patients, *P* values were calculated using a chi-square test for allele frequencies among 426,245 SNPs.

A quantile–quantile plot of the distribution of test statistics for the comparison of allele frequencies in early- and jaundice-stage PBC patients revealed that the inflation factor lambda was 1.014 for all tested SNPs (Supplementary Figure 2). Figure 1 shows a genome-wide view of the single-point association data based on allele frequencies in a comparison between 150 jaundice-stage and 975 early-stage PBC patients. Table 2 shows 12 SNPs with *P* < 5 × 10^−5^ in the GWAS. The SNP rs13720 (odds ratio [OR] = 2.15, 95% confidence interval [CI] = 1.58–2.94, *P* = 7.62 × 10^−7^), which is located in the 3′UTR of *cathepsin Z* (*CTSZ*), showed the strongest association with progression to jaundice stage in PBC.

**Table 2:** Association tests of twelve candidate SNPs with *P* < 5 × 10^−5^ in the GWAS.

Marker Information					GWAS stage				Validation stage			
					Statistics		MAF		Statistics		MAF	
rs ID	Chr	Position (hg19)	Nearest Gene	Minor allele	*P* value	OR	Jaundice-stage (n = 150)	Early-stage (n = 975)	*P* value	OR	Jaundice-stage (n = 173)	Early-stage (n = 1,202)
rs1600683	4	35175065	*ARAP2*	C	1.83E-05	1.71	0.460	0.333	2.66E-05	1.65	0.457	0.337
rs9322354	6	152382014	*ESR1*	G	4.63E-06	1.78	0.427	0.295	6.97E-05	1.62	0.407	0.298
rs1122661	7	35441980	*HERPUD2*	C	3.70E-05	1.95	0.197	0.111	3.68E-04	1.75	0.188	0.117
rs13298004	9	90234456	*DAPK1*	T	9.44E-06	1.79	0.353	0.234	2.65E-05	1.69	0.349	0.241
rs985079	10	29370208	*LYZL1*	G	2.51E-05	1.75	0.330	0.219	1.77E-04	1.62	0.315	0.221
rs9530012	13	73037479	*MZT1*	A	2.29E-05	1.76	0.327	0.216	1.92E-04	1.62	0.315	0.221
rs1958976	14	40242642	*LRFN5*	A	4.95E-06	2.74	0.099	0.039	2.10E-04	2.22	0.092	0.044
rs6498342	16	12707035	*CPPED1*	C	1.69E-05	0.44	0.104	0.210	2.66E-04	0.52	0.118	0.204
rs12953610	18	5288695	*ZFP161*	G	3.16E-05	2.05	0.164	0.088	2.96E-05	2.02	0.155	0.084
rs12479566	20	57564448	*NELFCD*	T	1.22E-06	2.13	0.211	0.112	1.31E-06	2.07	0.205	0.111
rs13720	20	57570568	*CTSZ*	G	7.62E-07	2.15	0.213	0.112	1.36E-07	2.15	0.211	0.110
rs9760	20	57571763	*CTSZ*	A	1.01E-06	1.98	0.283	0.166	1.02E-06	0.53	0.282	0.172

*P* values were calculated using a chi-square test for allele frequencies in the GWAS samples and all 1,375 samples.MAF: minor allele frequency.

*P* value of Pearson’s chi-square test for the allelic model.

Odds ratio (OR) of minor allele from two-by-two allele frequency table.

### Validation analysis and high-density association mapping

To validate the associations of the SNPs identified by the GWAS, we performed association tests of the 12 candidate SNPs using a total of 1,375 samples (173 jaundice-stage and 1,202 early-stage PBC patients), including the GWAS set of 1,125 samples and a replication set of 250 samples (23 jaundice-stage and 227 early-stage PBC patients) (Table 1). Although none of the 12 SNPs reached genome-wide significance (*P* = 1.17 × 10^−7^) in this validation analysis, SNP rs13720 exhibited a borderline significant association with progression to jaundice stage (OR = 2.15, 95% CI = 1.61–2.87, *P* = 1.36 × 10^−7^, Table 2).

**Figure 2:**
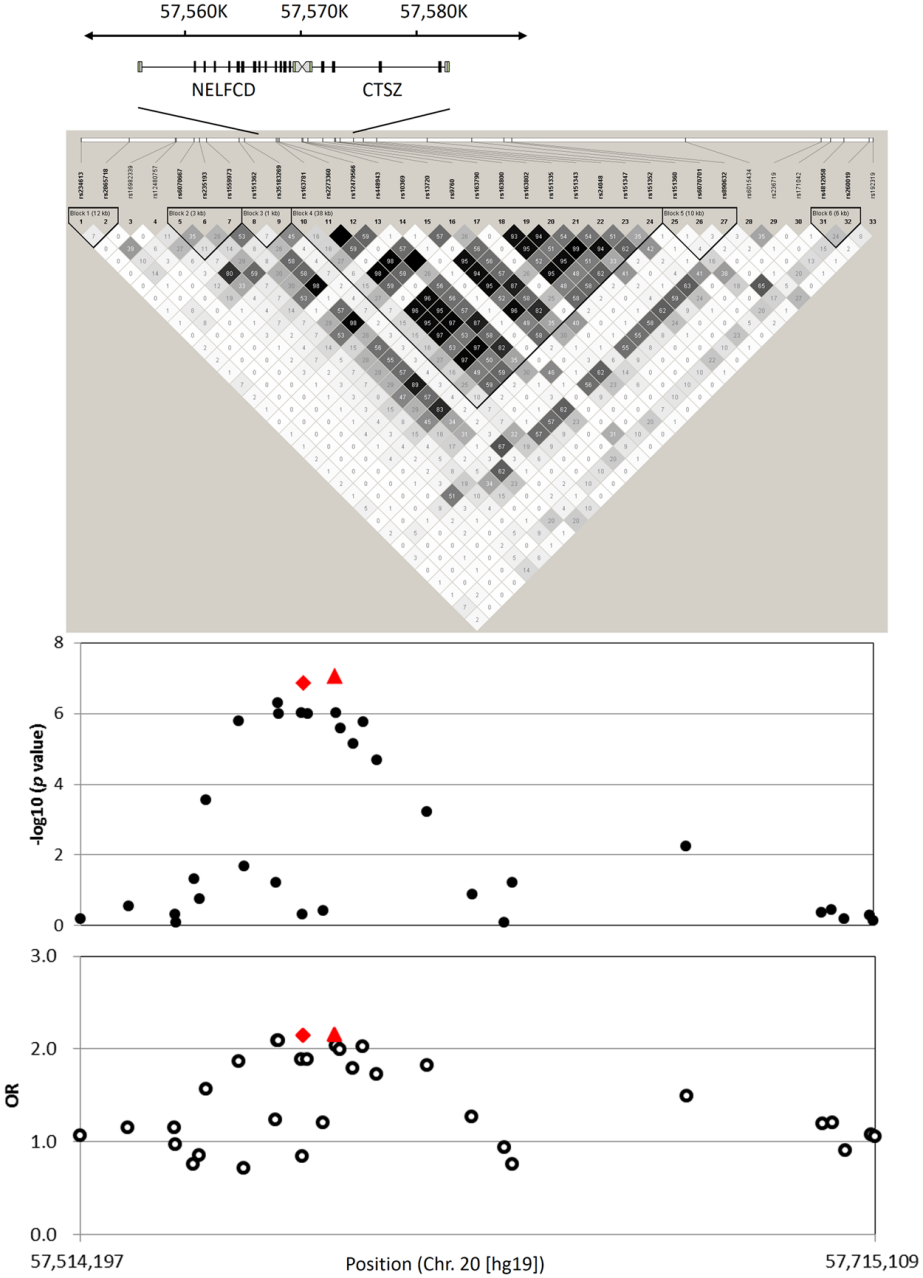
High-density association mapping at *NELFCD* and *CTSZ* loci. The top panel shows estimates of pairwise r^2^ for 33 SNPs used in the high-density association mapping at *NELFCD* and *CTSZ* loci (chr 20, nucleotide positions 57514197–57715109, hg19) using a total of 1,375 samples including 173 jaundice-stage and 1,202 early-stage PBC patients. The bottom panel shows *P* values (●) and OR (○) based on chi-square tests for the allelic model. Red diamond () and red triangle () show rs13720 and rs163800, respectively.

SNP rs13720 is located in a large linkage disequilibrium (LD) block including *negative elongation factor complex member C/D* (*NELFCD*) and *CTSZ*. Therefore, we performed high-density association mapping using a total of 1,375 samples by selecting 33 tagging SNPs in these loci (Fig. 2). In this analysis, an intronic SNP of *CTSZ*, rs163800, but not rs13720, exhibited the most significant association with progression to jaundice stage (OR = 2.16, 95% CI = 1.62–2.87, *P* = 8.57 × 10^−8^, Supplementary Table 2).

#### In silico analysis of CTSZ and NELFCD genes

Although the top hit, SNP rs163800, was predicted to have minimal binding evidence in the Regulome DB database, four genetic variants (rs3746703, rs151335, rs24048, and rs151336) surrounding rs163800 in strong LD (r^2^ > 0.8) were predicted to be located in transcriptional regulatory elements, which were identified based on DNase hypersensitivity cluster analyses, prediction of binding sites of transcription factors, and significant associations in eQTL analysis (Supplementary Table 3). Additionally, rs1043219 (located in the 3′UTR of *NELFCD*) and rs13720 were predicted to have certain biological effects within microRNA binding sites (Supplementary Table 4). Therefore, these polymorphisms, which are in strong LD with rs163800, may affect the gene expression levels of *CTSZ* and *NELFCD*.

**Figure 3:**
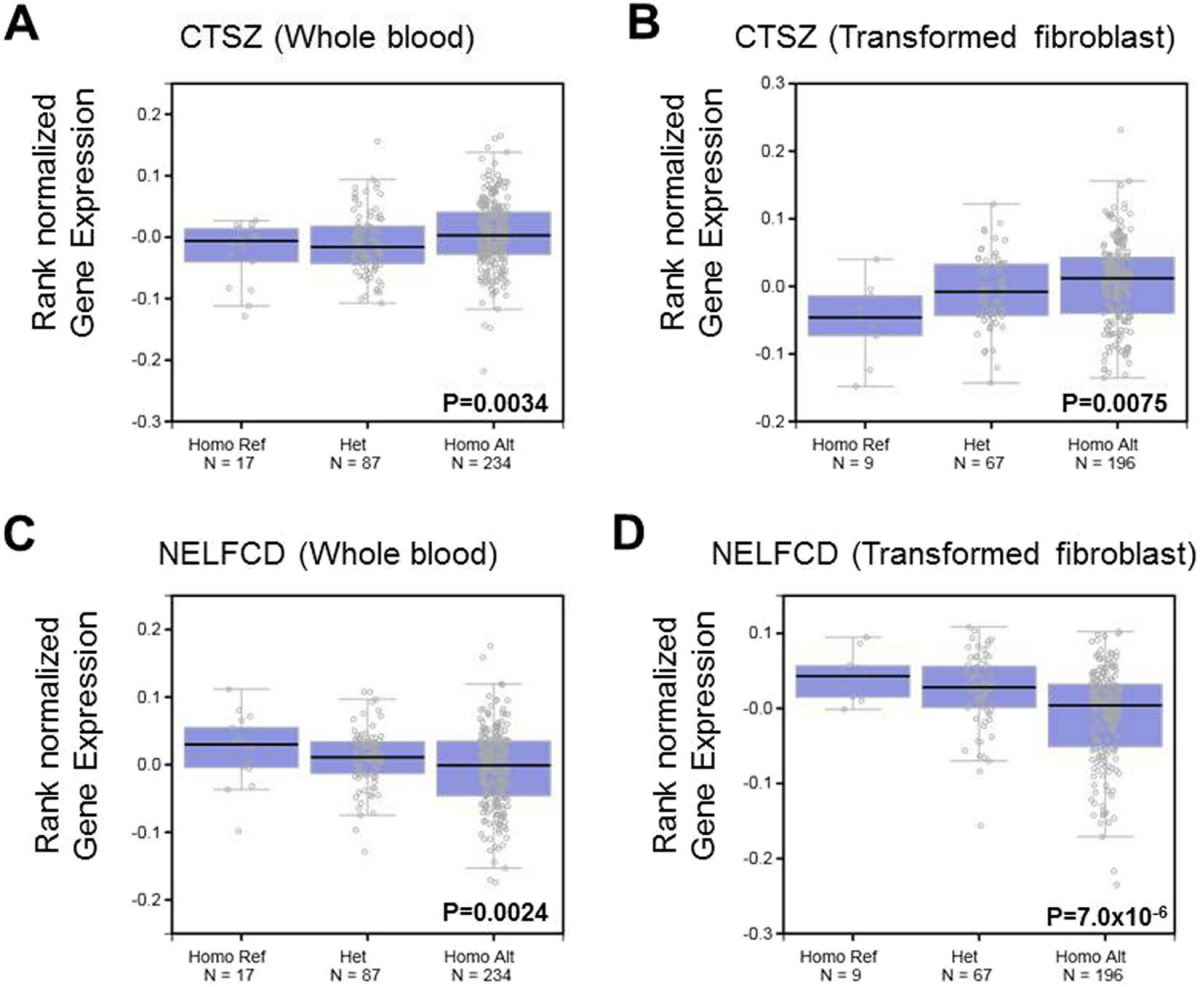
Endogenous mRNA expression levels of *CTSZ* and *NELFCD* as a function of rs163800 genotype in whole blood (**A**,**C**) and transformed fibroblast (**B**,**D**) Endogenous *CTSZ* and *NELFCD* expression data were extracted from the GTEx portal database. Risk (T) and non-risk alleles (**C**) of rs163800 for jaundice-stage progression in PBC are shown as reference and alteration alleles, respectively. Ref, reference; Het, heterogeneous; Homo, homogenous; Alt, alteration.

To assess the biological effects of these variants on mRNA expression, we compared the endogenous expression levels of *CTSZ* and *NELFCD* among rs163800 genotypes using the GTEx portal database. Because endogenous CTSZ and NELFCD proteins are abundantly expressed (Supplementary Figure 3), we extracted data from whole blood, transformed fibroblasts, liver, spleen, and EBV-transformed lymphocytes from the GTEx portal database. Among these organs, whole blood and transformed fibroblasts exhibited significant associations between mRNA levels and rs163800 genotype (*CTSZ*, whole blood: *P* = 0.0034; *CTSZ*, transformed fibroblast: *P* = 0.0075; *NELFCD*, whole blood: *P* = 0.0024; *NELFCD*, transformed fibroblast: *P* = 7.0 × 10^−6^, Fig. 3). The risk allele of rs163800 for jaundice-stage progression was associated with elevated *NELFCD* and reduced *CTSZ* mRNA levels in both tissue types. Although the associations in liver, spleen, and EBV-transformed lymphocytes did not reach statistical significance, probably due to the small sample size, the patterns in these three tissues resembled those in whole blood and transformed fibroblasts (Supplementary Figure 4). These results suggested that expression of both *CTSZ* and *NELFCD* is regulated based on the genotypes of rs163800 or other polymorphisms in strong LD with rs163800.

#### Multivariate analysis of anti-nuclear antibodies and CTSZ SNP for jaundice-stage progression in PBC

We previously reported that anti-gp210 and anti-centromere antibodies are risk factors for progression to jaundice stage and late stage without jaundice, respectively, in PBC^4^. Hence, we performed multivariate analysis (logistic regression analysis) to determine whether the *CTSZ* SNP is a risk factor independent of anti-nuclear antibodies for jaundice-stage progression in PBC. Consistent with our previous report (4), positivity for anti-gp210 antibodies was a strong risk factor for jaundice-stage progression (OR = 3.04, 95% CI = 2.15–4.31, *P* = 1.01 × 10^−9^). In addition, the risk allele of *CTSZ* SNP, rs163800, was a significant risk factor for jaundice-stage progression (OR = 2.22, 95% CI = 1.63–3.02, *P* = 8.77 × 10^−7^), independent of positivity for anti-gp210 antibodies (Table 3).

**Table 3:** Multivariate analysis for progression to jaundice stage.

Factor	*P* value	OR	95% CI
*CTSZ* rs613800	8.77 × 10^−7^	2.22	1.63–3.02
Anti-gp210 antibody positive	1.01 × 10^−9^	3.04	2.15–4.31
Anti-centromere antibody positive	0.07	0.66	0.41–1.05

## Discussion

GWAS and subsequent validation studies using a total of 1,375 samples (173 jaundice-stage and 1,202 early-stage Japanese PBC patients) identified a strong association between rs13720, located the *NELFCD/CTSZ* locus, and progression to jaundice-stage in PBC. In high-density association mapping using selected tagging SNPs from the *NELFCD/CTSZ* loci, SNP rs163800, located in an intronic region of *CTSZ*, exhibited a significant association even after Bonferroni correction. This SNP was confirmed as a risk factor for jaundice-stage progression independent of anti-gp210 antibody status. Moreover, *in silico* functional analysis suggested that expression levels of *CTSZ* and *NELFCD* may be influenced by the genotypes of rs163800 or variants in strong LD with rs163800. On the other hand, SNPs in these *NELFCD-CTSZ* loci did not show any association with the development of PBC (Supplementary Table 5), indicating that the mechanism of PBC-progression to jaundice stage is distinct from that of PBC-development.

Previous studies showed that *CTSZ* is involved in the development of neurodegenerative disorders, Huntington’s disease and other polyglutamine diseases, multiple sclerosis, and gastric and prostate cancer; furthermore, variants in this gene are associated with susceptibility to tuberculosis^23–25^. In this study, we demonstrated that *CTSZ* variants are associated with progression to jaundice stage in PBC, mediated through a reduction in *CTSZ* mRNA levels. CTSZ is a member of the cysteine cathepsins, which are mainly localized in lysosomes, and has exopeptidase activity. CTSZ regulates various cellular physiological functions, including adhesion, migration, invasion, and maturation of immune cells^26^. Therefore, we postulate that the risk allele of *CTSZ* might influence the regulation of cellular and/or immune functions through altered cleavage of various molecules, including self and exogenous proteins, leading to progression to jaundice stage in PBC.

NELFCD, which is also referred to as trihydrophobin 1, is an essential component of the NELF complex, which negatively regulates the elongation of transcription by RNA polymerase II^27^. NELFCD is expressed in a wide range of tissues including heart, brain, lung, liver, kidney, and prostate^28^. NELFCD inhibits cell migration and cell-cycle progression by negatively regulating MAPK signal transduction^29,30^. In addition, NELFCD represses estrogen alpha–mediated transactivation and androgen signal transduction, and its expression level is negatively correlated with progression and metastasis of breast cancer^28,31,32^. A previous report showed that elevated and reduced ER alpha levels in cholangiocytes are associated with aberrant cholangiocyte proliferation in both early- and late-stage PBC and ductopenia in end-stage PBC, respectively^33^. Collectively, these reports suggest that NELFCD plays a role in progression to jaundice stage in PBC via regulation of MAPK and hormonal signaling pathways.

Several host factors, including proteins encoded by the *MDR3*, *ITGAV*, *CTLA4*, *CYP7A1*, *HNF4A*, *PPARGC1A*, and *OCT-1* loci, have been reported to be associated with PBC progression in the Japanese population^19–22^. However, these factors were identified by candidate gene approaches, and results are not robust. Among these genes, only one (*PPARGC1A*) exhibited a significant association (*P* < 0.05) with disease progression to jaundice stage in our GWAS (Supplementary Table 5). This observation indicates that PPARGC1, a transcriptional activator of *CYP7A1*, is involved in disease progression of PBC via regulation of bile acid synthesis.

Positivity for anti-gp210 antibodies is a strong risk factor for jaundice-stage progression in PBC^4^. Recently, the UK-PBC and GLOBE scoring systems were proposed as practical methods to predict long-term outcome of PBC^5,6^. In these scoring systems, serum levels of biochemical markers such as albumin, bilirubin, alkaline phosphatase, and platelet numbers 1 year after the start of UDCA treatment are used as predictive factors. In a Chinese population, these scoring systems were further optimized by incorporation of anti-gp210 antibody positivity into the predictive factors^34^. In this study, we identified *CTSZ* SNP as a novel risk factor, independent of positivity for anti-gp210 antibodies, for jaundice-stage progression in PBC. The scoring system for predicting long-term outcome is currently under investigation by incorporating CTSZ SNP as a novel risk factor in Japanese patients with PBC.

Estimated risk reduction and transplantation by treatment with bezafibrate was reported very recently in patients with PBC by application of UK-PBC and Global PBC risk scores to BEZURO trial (Christophe Corpechot *et al*. New England Journal of Medicine, 2018 in press). In accordance with this results, the number of PBC patients who progressed to jaundice stage and underwent liver transplantation has been decreasing in Japan after the introduction of bezafibrate for the treatment of PBC, although a large randomized control studies are ongoing to validate the long-term effect of bezafibrate in Japanese PBC patients. Therefore, the role of the risk allele in *NELFCD* and *CTSZ loci* for jaundice-stage progression is also an important question to be answered in the ongoing prospective study under treatment with bezafibrate.

In conclusion, our GWAS identified *NELFCD* and *CTSZ* as novel loci associated with progression to jaundice stage in PBC. Functional SNPs at these loci may be involved in progression to jaundice stage via alteration of both *NELFCD* and *CTSZ* mRNA levels. Although further studies are needed to validate these observations in other independent cohorts, including patients of different ethnicities, our findings will provide novel insights into the genetic architecture of PBC progression to jaundice stage.

## Materials and Methods

### Ethics statement

All study protocols conform to the relevant ethical guidelines, as reflected in the *a priori* approval by the ethics committees of Nagasaki Medical Center and all institutes and hospitals that participated in this collaborative study. Written informed consent was obtained from all patients who participated in this study. All samples were anonymized and apart from the respective data. All methods applied in this study were carried out in accordance with the approved guidelines.

### Genomic DNA samples and clinical data

Genomic DNA samples from 1,375 Japanese PBC patients were collected by members of the Japan PBC-GWAS Consortium, consisting of 31 hospitals participating in the National Hospital Organization (NHO) Study Group for Liver Disease in Japan (NHOSLJ) and 24 university hospitals participating in gp210 Working Group in Intractable Liver Disease Research Project Team of the Japanese Ministry of Health and Welfare. Patients were diagnosed with PBC if they met at least two of the following three criteria: elevation of cholestatic liver enzymes, positive serum anti-mitochondrial antibodies (AMA), and typical histological findings of liver biopsy specimens. The overlap patients with autoimmune hepatitis was excluded based on the liver biopsy, episodes of high serum titers of ALT ≥200 mIU/ml, and response to prednisolone (0.5 to 1.0 mg/Kg body weight).

The clinical stages for PBC are defined as follows: early stage, no signs indicating portal hypertension or liver cirrhosis; late stage without jaundice, late stage with signs of portal hypertension or liver cirrhosis but without persistent jaundice; jaundice stage, late stage with persistent presence of jaundice, total bilirubin >2 mg/dL. The presence of portal hypertension or liver cirrhosis was diagnosed by ultrasound sonography, computed tomography, and esophago-gastroscopy. In order to efficiently identify genetic factors associated with progression to jaundice stage, we excluded 282 PBC patients in late stage without jaundice at the end of observation; approximately 12.0% of these PBC patients in late stage without jaundice potentially progress to jaundice stage in the future according to our observation that 15 out of 122 PBC patients (12.2%) at late-stage without jaundice progressed to jaundice stage, whereas 15 out of 1028 early-stage PBC patients (1.4%) progressed to jaundice stage and 90 out of 1028 early-stage PBC patients (8.8%) progressed to late stage without jaundice. Thus, of 1,375 Japanese PBC patients, 173 were diagnosed as jaundice-stage, and the remaining 1,202 as early-stage at the end of observation (Table 1).

The 173 jaundice-stage patients had the following clinical characteristics at the end of observation: 153 (88.4%) female; age, 27–76 years (median, 51 years); AMA positivity, 77.9%; glycoprotein (gp)−210 antibody positivity, 58.7%; centromere protein (CENP) antibody positivity, 15.1%; observation period, range 0–25 years, median 6.0 years; treatment, ursodeoxycholic acid only 56.0%, ursodeoxycholic acid + bezafibrate 25.6%, ursodeoxycholic acid + bezafibrate + prednisolone 8.0%. The treatment response one to two years after the initiation of ursodeoxycholic acid or ursodeoxycholic acid + bezafibrare was poor (ALP and/or ALT value > 1.5 upper limit normal) in 35 out of 44 patients (35/44 = 79.5%). For the 1,202 early-stage patients at the end of observation: 1,053 (87.6%) female; age, 23–89 years (median, 60 years); AMA positivity, 84.1%; gp210 antibody positivity, 20.4%; CENP antibody positivity, 23.6%; observation period, range 2–35 years, median 6.0 years; treatment. ursodeoxycholic acid only 68.2.0%, ursodeoxycholic acid + bezafibrate 24.2%, ursodeoxycholic acid + bezafibrate + prednisolone 2.2%. The treatment response one to two years after the initiation of ursodeoxycholic acid or ursodeoxycholic acid + bezafibrare was poor in 59 out of 873 patients (59/873 = 6.8%).

Genomic DNA was extracted from whole peripheral blood of 1,375 Japanese PBC patients using the QIAamp DNA Blood Midi Kit (Qiagen, Tokyo, Japan). One microgram of purified genomic DNA was dissolved in 100 µl of TE buffer (pH 8.0) (Wako, Osaka, Japan), followed by storage at −20 °C until use.

### GWAS

Two hundred nanograms of purified genomic DNA from each of 1,125 Japanese PBC patients (including 150 jaundice-stage and 975 early-stage patients) were genotyped as previously described^16^ using the Affymetrix Axiom Genome-Wide ASI 1 Array. SNP filtering for statistical analysis is summarized in Supplementary Table 1.

### Validation analysis and high-density association mapping

To validate the associations of SNPs identified by the GWAS and to carry out high-density association mapping in a genetic region including *NELFCD*-*CTSZ* locus, 12 and 33 SNPs, respectively, were genotyped in a total of 1,375 samples using the DigiTag2^35^ and custom TaqMan SNP genotyping assays (Applied Biosystems, Foster City, CA, USA) on a LightCycler 480 Real-time PCR system (Roche, Mannheim, Germany). For the high-density association mapping, 33 tagging SNPs were selected from a region of approximately 200 kb (chr20, nucleotide positions 57514197–57715109, hg19) based on haplotype blocks estimated from Japanese HapMap data using the Haploview software (Table 2).

### Databases

Predictions of miRNA binding to *CTSZ* rs13720 and *NELFCD* rs1043219 were obtained from FuncPred (https://snpinfo.niehs.nih.gov/snpinfo/snpfunc.html) and PolymiRTS (http://compbio.uthsc.edu/miRSNP/). Predictions of the locations of the variants in transcriptional regulatory elements were obtained from the RegulomeDB database (http://www.regulomedb.org/) and UCSC genome browser (http://genome.ucsc.edu/cgi-bin/hgGateway). Data regarding the correlation between rs163800 genotype and *CTSZ* or *NELFCD* expression were obtained from the GTEx portal database (http://gtexportal.org/home/).

### Statistical analysis

For the GWAS and validation analysis, *P* values, ORs, and CIs for the association between SNPs and disease progression in PBC patients were assessed by chi-squared test with a two-by-two contingency table for the allelic model. To avoid false-positive results due to multiple testing, significance levels for the GWAS and validation stages were adjusted based on the number of tested SNPs, i.e., *P* = 1.17 × 10^−7^ (0.05/426,245) and *P* = 1.52 × 10^−3^ (0.05/33), respectively. Multivariate logistic regression analysis was performed using gp-210 antibody positivity and CENP antibody positivity as covariates.

## Electronic supplementary material

Supplementary Information
