# Supplementary material for: NELFCD and CTSZ loci are associated with jaundice-stage progression in primary biliary cholangitis in the Japanese population
Source: Sci Rep. 2018 May 23;8:8071. doi: 10.1038/s41598-018-26369-6 (PMC5966418; doi:10.1038/s41598-018-26369-6)
Supplement: Supplementary file 1 — Supplementary Information [file 41598_2018_26369_MOESM1_ESM.docx]

**Supplementary Information**

NELFCD and CTSZ loci are associated with jaundice-stage progression in primary biliary cholangitis in the Japanese population

Nao Nishida, Yoshihiro Aiba, Yuki Hitomi, Minae Kawashima, Kaname Kojima, Yosuke Kawai, Kazuko Ueno, Hitomi Nakamura, Noriyo Yamashiki, Tomohiro Tanaka, Sumito Tamura, Akira Mori, Shintaro Yagi, Yuji Soejima, Tomoharu Yoshizumi, Mitsuhisa Takatsuki, Atsushi Tanaka, Kenichi Harada, Shinji Shimoda, Atsumasa Komori, Susumu Eguchi, Yoshihiko Maehara, Shinji Uemoto, Norihiro Kokudo, Masao Nagasaki, Katsushi Tokunaga, *Minoru Nakamura

Supplementary Figure 1. Principal component analysis of 1,125 GWAS and 265 HapMap samples (43 JPT, 40 CHB, 91 YRI, and 91 CEU samples).

(A) All groups. (B) Focused on Asian samples (JPT and CHB).


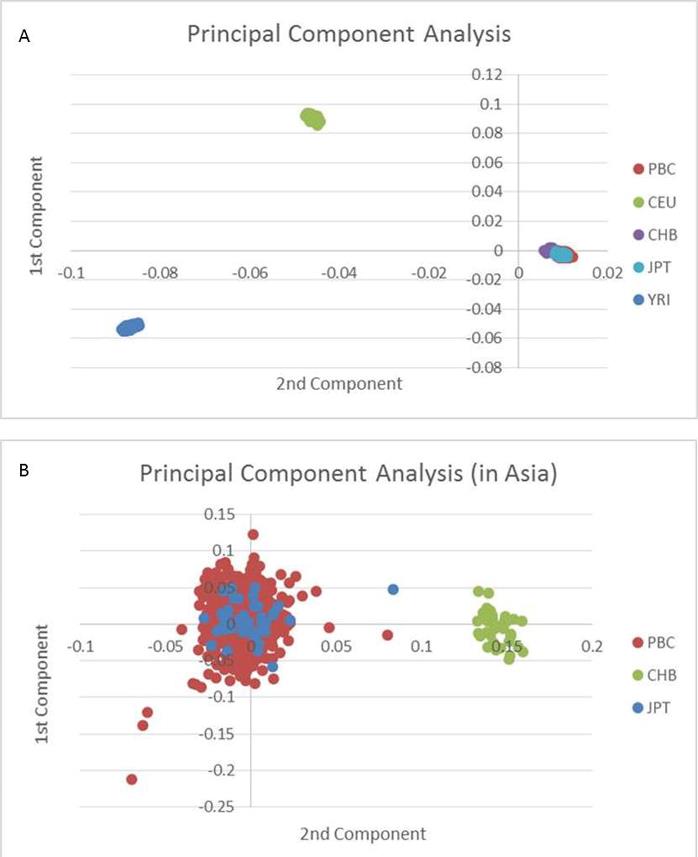


Supplementary Figure 2. Quantile–quantile plot of the allele-based chi-square test statistics for the GWAS results. The dots represent the *P* values of each SNP that passed the quality control filters. The inflation factor λ was estimated to be 1.014 for all SNPs tested.


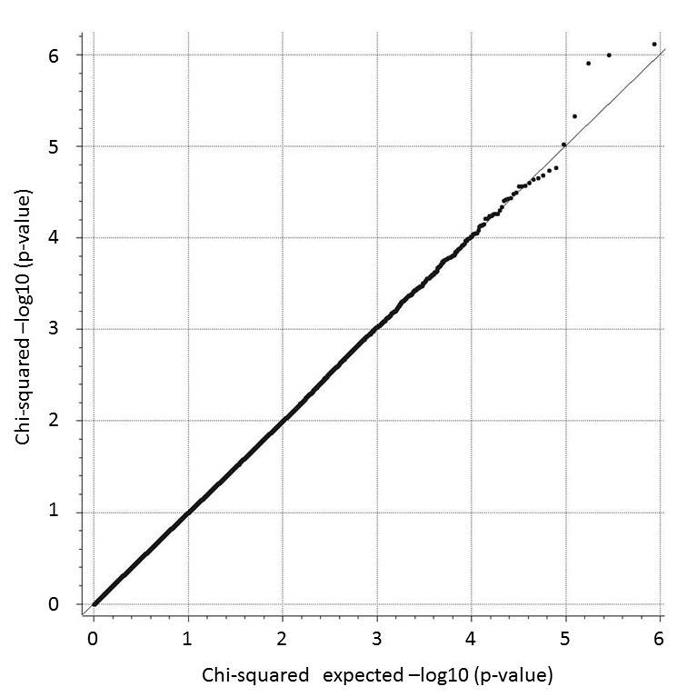


Supplementary Figure 3. Endogenous protein levels of CTSZ (A) and NELFCD (B) in various tissues and cell types.


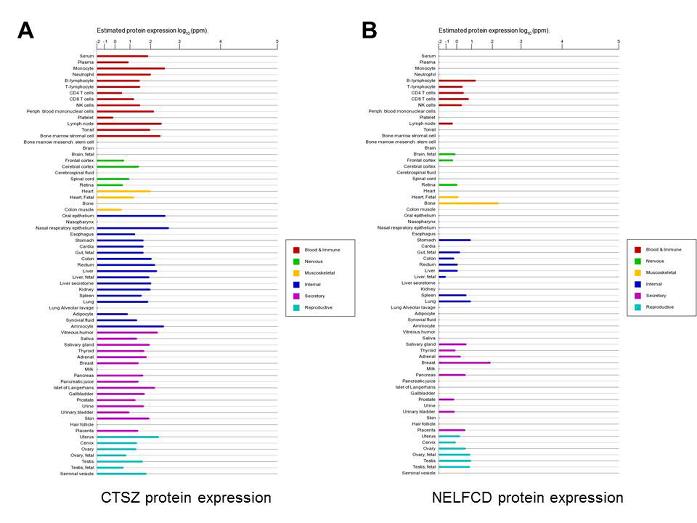


Supplementary Figure 4. Endogenous mRNA levels of *CTSZ* and *NELFCD* as a function of rs163800 genotype in liver (A and D), spleen (B and E), and EBV-transformed lymphocytes (C and F). Endogenous *CTSZ* and *NELFCD* expression data were extracted from the GTEx portal database.


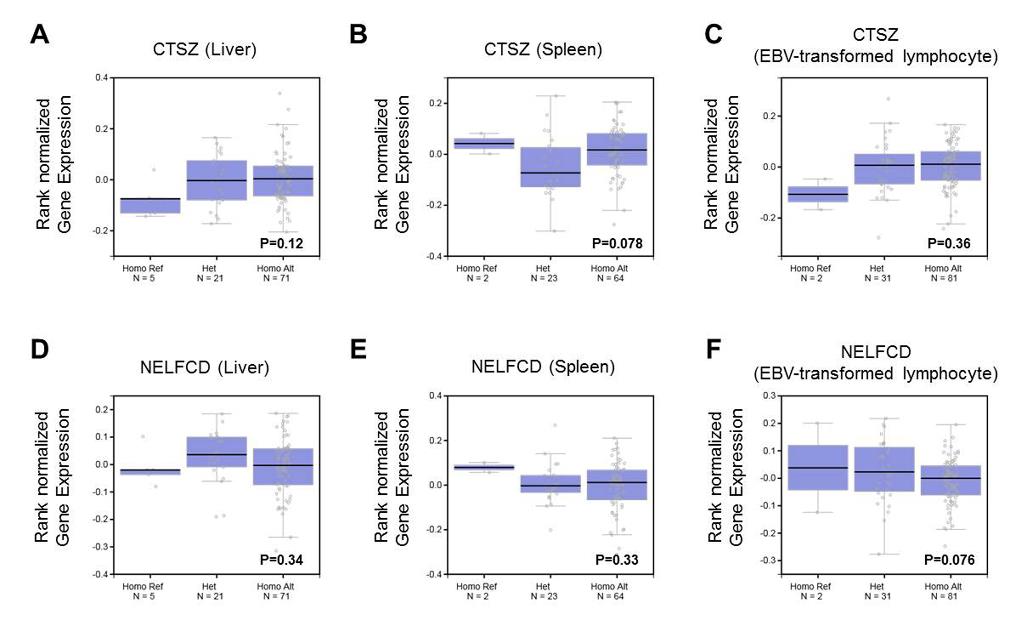


Supplementary Table 1. SNP filtering for statistical analysis

| QC Parameter | Threshold | # Excluded SNP |
| --- | --- | --- |
|  |  | Autosomal Chromosomes |
| SNP call rate | < 0.95 | 8,222 |
| MAF | < 0.05 | 146,776 |
| HWEp | < 0.001 | 6,179 |

Supplementary Table 2. High-density association mapping in a chromosomal region including the *NELFCD*–*CTSZ* locus. From all 1,375 Japanese samples including 173 jaundice-stage and 1,202 early-stage PBC patients, *P* values were calculated using a chi-square test for allele frequencies.

| Marker information | | | | Statistics | | MAF | |
| --- | --- | --- | --- | --- | --- | --- | --- |
| rs ID | Chr | Position (hg19) | Minor  allele | *P* value^a^ | OR^b^ | Jaundice-stage | Early-  stage |
|  |  |  |  |  |  | (n = 173) | (n = 1,202) |
| rs234613 | 20 | 57514197 | G | 0.659 | 1.07 | 0.20 | 0.19 |
| rs2865718 | 20 | 57526412 | G | 0.275 | 1.15 | 0.27 | 0.24 |
| rs16982339 | 20 | 57538175 | G | 0.495 | 1.16 | 0.08 | 0.07 |
| rs12480757 | 20 | 57538331 | T | 0.816 | 0.97 | 0.35 | 0.36 |
| rs6070667 | 20 | 57542883 | T | 0.047 | 0.76 | 0.23 | 0.28 |
| rs235193 | 20 | 57544274 | A | 0.180 | 0.86 | 0.45 | 0.49 |
| rs1559973 | 20 | 57546046 | T | 2.82E-04 | 1.57 | 0.33 | 0.24 |
| rs151362 | 20 | 57554311 | C | 1.69E-06 | 1.86 | 0.28 | 0.17 |
| rs35183269 | 20 | 57555717 | C | 0.020 | 0.72 | 0.20 | 0.26 |
| rs163781 | 20 | 57563765 | A | 0.061 | 1.24 | 0.48 | 0.43 |
| rs2273360 | 20 | 57564070 | T | 5.02E-07 | 2.09 | 0.20 | 0.11 |
| rs12479566 | 20 | 57564448 | T | 1.02E-06 | 2.09 | 0.21 | 0.11 |
| rs448943 | 20 | 57570238 | A | 9.75E-07 | 1.89 | 0.28 | 0.17 |
| rs10369 | 20 | 57570456 | T | 0.472 | 0.85 | 0.07 | 0.08 |
| rs13720 | 20 | 57570568 | G | 1.36E-07 | 2.15 | 0.21 | 0.11 |
| rs9760 | 20 | 57571763 | A | 1.02E-06 | 1.89 | 0.28 | 0.17 |
| rs163790 | 20 | 57575594 | C | 0.379 | 1.21 | 0.08 | 0.07 |
| rs163800 | 20 | 57578508 | T | 8.57E-08 | 2.16 | 0.22 | 0.11 |
| rs163802 | 20 | 57578852 | C | 9.76E-07 | 2.04 | 0.21 | 0.11 |
| rs151335 | 20 | 57579950 | A | 2.70E-06 | 1.99 | 0.20 | 0.11 |
| rs151343 | 20 | 57583298 | C | 7.38E-06 | 1.79 | 0.29 | 0.19 |
| rs24048 | 20 | 57585789 | T | 1.77E-06 | 2.02 | 0.20 | 0.11 |
| rs151347 | 20 | 57589174 | C | 2.07E-05 | 1.73 | 0.29 | 0.19 |
| rs151352 | 20 | 57601995 | A | 0.001 | 1.83 | 0.13 | 0.08 |
| rs151360 | 20 | 57613373 | C | 0.131 | 1.27 | 0.16 | 0.13 |
| rs6070701 | 20 | 57621455 | C | 0.795 | 0.95 | 0.10 | 0.11 |
| rs890632 | 20 | 57623551 | G | 0.060 | 0.76 | 0.18 | 0.22 |
| rs6015434 | 20 | 57667619 | T | 0.006 | 1.49 | 0.20 | 0.15 |
| rs236719 | 20 | 57701982 | T | 0.421 | 1.19 | 0.08 | 0.06 |
| rs171042 | 20 | 57704379 | T | 0.367 | 1.20 | 0.09 | 0.08 |
| rs4812058 | 20 | 57707719 | C | 0.628 | 0.91 | 0.10 | 0.11 |
| rs260019 | 20 | 57714225 | C | 0.517 | 1.08 | 0.36 | 0.34 |
| rs192319 | 20 | 57715109 | T | 0.717 | 1.06 | 0.15 | 0.15 |

MAF: minor allele frequency

^a^*P* value of Pearson’s chi-square test for allelic model.

^b^Odds ratio (OR) of minor allele from two-by-two allele frequency table.

Supplementary Table 3. Functional prediction of variants showing strong LD with rs163800 (r^2^ > 0.8)

| Position (hg19, Chr20) | r^2^ | D' | rs ID | Ref | Alt | Genes | Location | Regulome DB^a^ | UCSC^b^ |
| --- | --- | --- | --- | --- | --- | --- | --- | --- | --- |
| 57554367 | 0.87 | -0.95 | rs3761260 | G | A | 1.9 kb 5' of *NELFCD* |  | 5 | × |
| 57556488 | 0.82 | -0.95 | rs3746703 | C | T | *NELFCD* | intronic | 4 | ○ |
| 57557544 | 0.9 | -0.95 | rs76168789 | T | C | *NELFCD* | intronic | 4 | × |
| 57557579 | 0.9 | -0.95 | rs73295298 | A | G | *NELFCD* | intronic | 4 | × |
| 57557652 | 0.9 | -0.95 | rs79087230 | G | C | *NELFCD* | intronic | 4 | × |
| 57558296 | 0.9 | -0.95 | rs112452363 | A | G | *NELFCD* | intronic | 5 | × |
| 57558382 | 0.9 | -0.95 | rs59251281 | T | G | *NELFCD* | intronic | No data | × |
| 57558481 | 0.9 | -0.95 | rs111769360 | G | A | *NELFCD* | intronic | No data | × |
| 57559630 | 0.9 | -0.95 | rs12480262 | A | G | *NELFCD* | intronic | 1f | × |
| 57559677 | 0.9 | -0.95 | rs12480267 | A | C | *NELFCD* | intronic | 5 | × |
| 57559842 | 0.9 | -0.95 | rs56979548 | G | A | *NELFCD* | intronic | 5 | × |
| 57560777 | 0.9 | -0.95 | rs41276958 | C | T | *NELFCD* | intronic | 5 | × |
| 57563409 | 0.94 | -0.97 | rs78194738 | C | T | *NELFCD* | intronic | 3a | × |
| 57563609 | 0.94 | -0.97 | rs75454359 | A | C | *NELFCD* | intronic | 2b | × |
| 57564070 | 0.95 | -0.98 | rs2273360 | C | T | *NELFCD* | synonymous | - | - |
| 57564448 | 0.95 | -0.98 | rs12479566 | C | T | *NELFCD* | intronic | 5 | × |
| 57565943 | 0.95 | 0.98 | rs163782 | G | C | *NELFCD* | intronic | 4 | × |
| 57567543 | 0.95 | 0.98 | rs184673 | A | G | *NELFCD* | intronic | 5 | × |
| 57567730 | 0.95 | 0.98 | rs163783 | A | C | *NELFCD* | intronic | 5 | × |
| 57569860 | 0.94 | -0.97 | rs1043219 | C | G | *NELFCD* | 3'-UTR | - | - |
| 57570568 | 0.94 | 0.97 | rs13720 | G | A | *CTSZ* | 3'-UTR | - | - |
| 57573320 | 0.95 | -0.98 | rs56775894 | TAAA | T | *CTSZ* | intronic | 6 | × |
| 57574463 | 0.98 | 1 | rs163787 | A | G | *CTSZ* | intronic | 2b | × |
| 57576174 | 0.88 | 1 | rs163791 | G | A | *CTSZ* | intronic | 5 | × |
| 57576180 | 0.98 | 1 | rs191490 | A | G | *CTSZ* | intronic | 5 | × |
| 57577505 | 0.98 | 1 | rs163795 | G | C | *CTSZ* | intronic | 3a | × |
| 57578508 | - | - | rs163800 | T | C | *CTSZ* | intronic | 5 | × |
| 57578614 | 1 | 1 | rs163801 | A | G | *CTSZ* | intronic | 5 | × |
| 57578852 | 0.95 | 1 | rs163802 | C | G | *CTSZ* | intronic | 5 | × |
| 57579950 | 0.97 | 0.98 | rs151335 | A | G | *CTSZ* | intronic | 2b | ○ |
| 57585789 | 0.92 | 0.97 | rs24048 | T | C | 3.5kb 5' of *CTSZ* |  | 4 | ○ |
| 57586953 | 0.92 | 0.97 | rs151336 | T | G | 4.7kb 5' of *CTSZ* |  | 3a | ○ |

^a^Functional prediction scores of each SNPs by RegulomeDB database.

^b^Probability of functional damage, as determined using the UCSC genome browser.

Supplementary Table 4. Prediction of miRNA binding to *CTSZ* rs13720 and *NELFCD* rs1043219

| **Gene** | **rs ID** | **Allele** | **Strand** | | **Forward Sequence** | | **miRNA** | | | **miRanda** | | **Sanger** | | **PolymiRTS** | |
| --- | --- | --- | --- | --- | --- | --- | --- | --- | --- | --- | --- | --- | --- | --- | --- |
| *CTSZ* | rs13720 | A | | - | | aGTACCACGCTGTCCTCGCCAt | | hsa-miR-1227 | ○ | |  | |  | |  |
|  | rs13720 | G | | - | | aGTGCCACGCTGTCCTCGCCAt | | hsa-miR-1227 | ○ | |  | | ○ | |  |
|  | rs13720 | A | | - | | gCCAGTACCACGCTGTCC | | hsa-miR-1825 | ○ | |  | |  | |  |
|  | rs13720 | G | | - | | gCCAGTGCCACGCTGTCC | | hsa-miR-1825 | ○ | |  | | ○ | |  |
|  | rs13720 | A | | - | | gAACACTCGCAGCCAGTACCa | | hsa-miR-543 | ○ | | ○ | |  | |  |
|  | rs13720 | G | | - | | gAACACTCGCAGCCAGTGCCa | | hsa-miR-543 | ○ | | ○ | |  | |  |
|  | rs13720 | A | | - | | cAGCCAGTACCACgctgtc | | hsa-miR-575 | ○ | | ○ | |  | |  |
|  | rs13720 | G | | - | | cAGCCAGTGCCACgctgtc | | hsa-miR-575 | ○ | | ○ | |  | |  |
|  | rs13720 | G | | - | | cTCGCAGCCAGTGCCACGCTGT | | hsa-miR-639 | ○ | |  | |  | |  |
|  | rs13720 | A | | - | | gCCAGTACCACGCTGTCCTCGCCA | | hsa-miR-770-5p | ○ | | ○ | | ○ | |  |
|  | rs13720 | G | | - | | gCCAGTGCCACGCTGTCCTCGCCA | | hsa-miR-770-5p | ○ | | ○ | |  | |  |
|  | rs13720 | A | | - | | CAGGAACACTCGCAGCCagta | | hsa-miR-873 | ○ | | ○ | |  | |  |
|  | rs13720 | G | | - | | CAGGAACACTCGCAGCCagtg | | hsa-miR-873 | ○ | | ○ | |  | |  |
|  | rs13720 | A | | - | | gCCAGTACcacgc | | hsa-miR-4712-5p |  | |  | | ○ | |  |
|  | rs13720 | A | | - | | gccAGTACCAcgc | | hsa-miR-624-5p |  | |  | | ○ | |  |
| *NELFCD* | rs1043219 | G | | + | | ggaggAGGTGGATGAGTTCTTTa | | hsa-miR-186 | ○ | |  | |  | |  |
|  | rs1043219 | C | | + | | gCTGGGAGGAGGtGGAtGAc | | hsa-miR-877 |  | |  | | ○ | |  |

| Supplementary Table 5. Association study for PBC (1381 PBCs and 1505 controls) reported elswhere^17^ (chr.20: 57,490,000-57,720,000 [hg19]). | | | | | | | |  |
| --- | --- | --- | --- | --- | --- | --- | --- | --- |
|  |  |  |  |  |  |  |  |  |
| Marker information | | | | Statistics | | MAF | |  |
| rs ID | Chr | Position (hg19) | Minor allele | P value^a^ | OR^b^ | PBC (n=1381) | control (n=1505) |  |
| rs151331 | 20 | 57492504 | A | 0.82 | 0.99 | 0.23 | 0.24 |  |
| rs47223 | 20 | 57496873 | A | 0.50 | 1.04 | 0.29 | 0.29 |  |
| rs2865718 | 20 | 57526412 | G | 0.97 | 1.00 | 0.25 | 0.25 |  |
| rs6026615 | 20 | 57528279 | A | 0.85 | 1.01 | 0.25 | 0.25 |  |
| rs12480757 | 20 | 57538331 | T | 0.93 | 1.00 | 0.37 | 0.37 |  |
| rs235189 | 20 | 57540782 | G | 0.94 | 0.99 | 0.16 | 0.16 |  |
| rs235193 | 20 | 57544274 | A | 0.34 | 1.05 | 0.49 | 0.48 |  |
| rs6100297 | 20 | 57546301 | T | 0.70 | 1.02 | 0.26 | 0.26 |  |
| rs6026629 | 20 | 57546540 | A | 0.05 | 0.88 | 0.24 | 0.26 |  |
| rs467040 | 20 | 57548029 | T | 0.99 | 1.00 | 0.45 | 0.45 |  |
| rs6070675 | 20 | 57552033 | C | 0.69 | 1.03 | 0.25 | 0.24 |  |
| rs12479566 | 20 | 57564448 | T | 0.21 | 1.11 | 0.12 | 0.11 |  |
| rs7341 | 20 | 57569816 | G | 0.78 | 1.02 | 0.43 | 0.42 |  |
| rs10369 | 20 | 57570456 | T | 0.49 | 1.07 | 0.08 | 0.07 |  |
| rs13720 | 20 | 57570568 | G | 0.20 | 1.11 | 0.12 | 0.11 |  |
| rs9760 | 20 | 57571763 | A | 0.99 | 1.00 | 0.18 | 0.18 |  |
| rs2295357 | 20 | 57572839 | T | 0.10 | 0.84 | 0.06 | 0.07 |  |
| rs6070692 | 20 | 57586374 | G | 0.28 | 0.90 | 0.07 | 0.08 |  |
| rs34524896 | 20 | 57594684 | T | 0.27 | 1.11 | 0.09 | 0.08 |  |
| rs6070697 | 20 | 57599402 | A | 0.45 | 0.94 | 0.11 | 0.11 |  |
| rs6070701 | 20 | 57621455 | C | 0.88 | 1.01 | 0.11 | 0.11 |  |
| rs890632 | 20 | 57623551 | G | 0.73 | 0.98 | 0.22 | 0.22 |  |
| rs6070710 | 20 | 57642182 | G | 0.92 | 0.99 | 0.11 | 0.11 |  |
| rs6026675 | 20 | 57653416 | T | 0.71 | 0.97 | 0.15 | 0.15 |  |
| rs6123849 | 20 | 57658783 | A | 0.62 | 0.96 | 0.15 | 0.15 |  |
| rs13036348 | 20 | 57678828 | T | 0.95 | 1.00 | 0.14 | 0.14 |  |
| rs236706 | 20 | 57691416 | C | 0.33 | 0.90 | 0.07 | 0.07 |  |
| rs236713 | 20 | 57695577 | G | 0.31 | 0.90 | 0.06 | 0.07 |  |
| rs6026720 | 20 | 57712324 | T | 0.30 | 0.90 | 0.06 | 0.07 |  |
| rs6026721 | 20 | 57712488 | G | 0.37 | 0.91 | 0.06 | 0.07 |  |
| rs260019 | 20 | 57714225 | C | 0.14 | 0.92 | 0.35 | 0.37 |  |
| MAF: minor allele frequency | | |  |  |  |  |  |  |
| a P value of Pearson’s chi-square test for allelic model. | | | | | |  |  |  |
| b Odds ratio (OR) of minor allele from two-by-two allele frequency table. | | | | | | |  |  |

| Supplementary Table 6. GWAS associations of SNPs in previously reported host factors | | | | | | | | | |
| --- | --- | --- | --- | --- | --- | --- | --- | --- | --- |
| Marker Information | | | | | Statistics | | MAF | |  |
| rs ID | Chr | Position | Gene | Minor allele | *P* value^a^ | OR^b^ | Jaundice-stage | Early-stage |  |
|  |  | (hg19) |  |  |  |  | (n = 150) | (n = 975) |  |
| rs397710720 | 2 | 187458514 | *ITGAV* | del | 0.237 | 1.22 | 0.162 | 0.137 |  |
| rs3754584 | 2 | 187459497 | *ITGAV* | G | 0.584 | 1.13 | 0.082 | 0.073 |  |
| rs9333288 | 2 | 187487160 | *ITGAV* | G | 0.311 | 1.19 | 0.160 | 0.138 |  |
| rs3754586 | 2 | 187491926 | *ITGAV* | T | 0.472 | 0.85 | 0.077 | 0.089 |  |
| rs10174098 | 2 | 187492742 | *ITGAV* | G | 0.272 | 1.18 | 0.235 | 0.207 |  |
| rs2887831 | 2 | 187541810 | *ITGAV* | C | 0.113 | 1.37 | 0.113 | 0.085 |  |
| rs231779 | 2 | 204734487 | *CTLA4* | C | 0.389 | 0.89 | 0.323 | 0.349 |  |
| rs3087243 | 2 | 204738919 | *CTLA4* | A | 0.753 | 0.96 | 0.243 | 0.252 |  |
| rs2279525 | 4 | 23794252 | *PPARGC1A* | C | 0.510 | 0.88 | 0.114 | 0.128 |  |
| rs3774923 | 4 | 23795064 | *PPARGC1A* | T | 0.843 | 0.97 | 0.200 | 0.205 |  |
| rs6821591 | 4 | 23797000 | *PPARGC1A* | C | 0.795 | 1.04 | 0.269 | 0.262 |  |
| rs12650562 | 4 | 23801187 | *PPARGC1A* | C | 0.937 | 0.99 | 0.467 | 0.469 |  |
| rs2932965 | 4 | 23805486 | *PPARGC1A* | A | 0.494 | 1.11 | 0.223 | 0.206 |  |
| rs3774920 | 4 | 23812638 | *PPARGC1A* | C | 0.839 | 0.97 | 0.206 | 0.211 |  |
| rs3755863 | 4 | 23815522 | *PPARGC1A* | T | 0.966 | 1.01 | 0.463 | 0.462 |  |
| rs8192678 | 4 | 23815662 | *PPARGC1A* | T | 0.986 | 1.00 | 0.460 | 0.459 |  |
| rs2970848 | 4 | 23817027 | *PPARGC1A* | G | 0.479 | 0.90 | 0.243 | 0.263 |  |
| rs2932971 | 4 | 23818884 | *PPARGC1A* | T | 0.365 | 0.76 | 0.043 | 0.056 |  |
| rs2970852 | 4 | 23821523 | *PPARGC1A* | T | 0.380 | 0.77 | 0.044 | 0.056 |  |
| rs2970853 | 4 | 23823519 | *PPARGC1A* | A | 0.304 | 1.15 | 0.299 | 0.270 |  |
| rs4619879 | 4 | 23834876 | *PPARGC1A* | C | 0.768 | 1.04 | 0.490 | 0.481 |  |
| rs10938964 | 4 | 23840088 | *PPARGC1A* | G | 0.622 | 1.06 | 0.490 | 0.475 |  |
| rs6448227 | 4 | 23848631 | *PPARGC1A* | T | 0.716 | 1.05 | 0.240 | 0.230 |  |
| rs7665116 | 4 | 23853011 | *PPARGC1A* | C | 0.502 | 0.91 | 0.270 | 0.289 |  |
| rs12374310 | 4 | 23854149 | *PPARGC1A* | C | 0.526 | 1.08 | 0.460 | 0.440 |  |
| rs6448228 | 4 | 23862142 | *PPARGC1A* | G | 0.293 | 1.14 | 0.425 | 0.393 |  |
| rs4452416 | 4 | 23863873 | *PPARGC1A* | G | 0.405 | 0.86 | 0.123 | 0.141 |  |
| rs4697426 | 4 | 23864809 | *PPARGC1A* | T | 0.229 | 1.20 | 0.223 | 0.194 |  |
| rs28579569 | 4 | 23865257 | *PPARGC1A* | C | 0.436 | 1.13 | 0.217 | 0.197 |  |
| **rs7656250** | **4** | **23866016** | ***PPARGC1A*** | **C** | **0.040** | **0.76** | **0.333** | **0.395** |  |
| rs10212638 | 4 | 23866115 | *PPARGC1A* | G | 0.374 | 1.25 | 0.067 | 0.054 |  |
| **rs7677000** | **4** | **23869454** | ***PPARGC1A*** | **T** | **0.036** | **1.38** | **0.207** | **0.158** |  |
| rs9996943 | 4 | 23871021 | *PPARGC1A* | C | 0.712 | 1.05 | 0.446 | 0.435 |  |
| rs12503529 | 4 | 23873828 | *PPARGC1A* | C | 0.738 | 0.93 | 0.100 | 0.106 |  |
| rs13131226 | 4 | 23874981 | *PPARGC1A* | C | 0.100 | 1.23 | 0.417 | 0.367 |  |
| rs6838600 | 4 | 23875311 | *PPARGC1A* | A | 0.280 | 1.15 | 0.453 | 0.419 |  |
| rs16874265 | 4 | 23877229 | *PPARGC1A* | A | 0.768 | 1.06 | 0.097 | 0.091 |  |
| rs13128633 | 4 | 23877918 | *PPARGC1A* | G | 0.388 | 1.14 | 0.205 | 0.184 |  |
| rs4469064 | 4 | 23883796 | *PPARGC1A* | G | 0.576 | 1.13 | 0.097 | 0.087 |  |
| rs12500214 | 4 | 23885568 | *PPARGC1A* | A | 0.674 | 1.08 | 0.143 | 0.134 |  |
| rs2946385 | 4 | 23886323 | *PPARGC1A* | T | 0.407 | 1.11 | 0.385 | 0.360 |  |
| rs2970873 | 4 | 23889781 | *PPARGC1A* | A | 0.232 | 1.16 | 0.413 | 0.377 |  |
| rs2970872 | 4 | 23889813 | *PPARGC1A* | G | 0.874 | 1.03 | 0.174 | 0.171 |  |
| rs3774902 | 4 | 23890782 | *PPARGC1A* | A | 0.148 | 0.82 | 0.307 | 0.349 |  |
| rs3822840 | 6 | 160554662 | *SLC22A1* | T | 0.101 | 1.36 | 0.130 | 0.099 |  |
| rs594709 | 6 | 160555754 | *SLC22A1* | G | 0.518 | 0.90 | 0.201 | 0.218 |  |
| rs3777392 | 6 | 160556642 | *SLC22A1* | T | 0.405 | 0.82 | 0.070 | 0.084 |  |
| rs3798168 | 6 | 160559807 | *SLC22A1* | A | 0.970 | 1.00 | 0.407 | 0.408 |  |
| rs2197296 | 6 | 160568997 | *SLC22A1* | A | 0.132 | 1.22 | 0.362 | 0.319 |  |
| rs9295123 | 6 | 160570114 | *SLC22A1* | A | 0.174 | 1.19 | 0.379 | 0.339 |  |
| rs622591 | 6 | 160579527 | *SLC22A1* | C | 0.557 | 1.08 | 0.450 | 0.432 |  |
| rs4148828 | 7 | 87048530 | *ABCB4* | G | 0.935 | 1.02 | 0.110 | 0.108 |  |
| rs45461491 | 7 | 87055721 | *ABCB4* | G | 0.624 | 0.91 | 0.130 | 0.141 |  |
| rs45488793 | 7 | 87066792 | *ABCB4* | C | 0.931 | 1.02 | 0.057 | 0.055 |  |
| rs31674 | 7 | 87068464 | *ABCB4* | T | 0.695 | 0.95 | 0.305 | 0.317 |  |
| rs31676 | 7 | 87069880 | *ABCB4* | T | 0.739 | 0.96 | 0.307 | 0.316 |  |
| rs17149608 | 7 | 87073594 | *ABCB4* | A | 0.950 | 1.01 | 0.277 | 0.275 |  |
| rs4148826 | 7 | 87074419 | *ABCB4* | C | 0.502 | 1.10 | 0.312 | 0.292 |  |
| rs4148822 | 7 | 87077996 | *ABCB4* | G | 0.330 | 1.15 | 0.273 | 0.247 |  |
| rs1202283 | 7 | 87082292 | *ABCB4* | A | 0.699 | 1.05 | 0.405 | 0.394 |  |
| rs17149644 | 7 | 87086218 | *ABCB4* | C | 0.687 | 1.06 | 0.303 | 0.292 |  |
| rs4148814 | 7 | 87091543 | *ABCB4* | C | 0.209 | 1.19 | 0.294 | 0.259 |  |
| rs4148812 | 7 | 87101407 | *ABCB4* | G | 0.665 | 0.94 | 0.300 | 0.312 |  |
| rs8192879 | 8 | 59403576 | *CYP7A1* | T | 0.523 | 1.09 | 0.303 | 0.284 |  |
| rs8192878 | 8 | 59404346 | *CYP7A1* | C | 0.716 | 1.09 | 0.077 | 0.071 |  |
| rs8192877 | 8 | 59404441 | *CYP7A1* | G | 0.445 | 1.20 | 0.073 | 0.062 |  |
| rs11786580 | 8 | 59405940 | *CYP7A1* | T | 0.428 | 1.17 | 0.107 | 0.092 |  |
| rs3747809 | 8 | 59406984 | *CYP7A1* | C | 0.782 | 0.93 | 0.057 | 0.061 |  |
| rs1457042 | 8 | 59410632 | *CYP7A1* | T | 0.494 | 1.09 | 0.356 | 0.336 |  |
| rs6996287 | 8 | 59411394 | *CYP7A1* | T | 0.239 | 1.16 | 0.466 | 0.429 |  |
| rs17755638 | 20 | 42987841 | *HNF4A* | C | 0.828 | 1.03 | 0.200 | 0.195 |  |
| rs4812829 | 20 | 42989267 | *HNF4A* | A | 0.236 | 1.16 | 0.487 | 0.450 |  |
| rs6031552 | 20 | 42989794 | *HNF4A* | A | 0.699 | 1.06 | 0.203 | 0.194 |  |
| rs6103716 | 20 | 42999630 | *HNF4A* | A | 0.309 | 0.88 | 0.390 | 0.421 |  |
| rs6073418 | 20 | 43000590 | *HNF4A* | T | 0.212 | 0.82 | 0.190 | 0.222 |  |
| rs6017335 | 20 | 43010825 | *HNF4A* | G | 0.529 | 0.92 | 0.373 | 0.392 |  |
| rs4812831 | 20 | 43018260 | *HNF4A* | A | 0.908 | 1.01 | 0.380 | 0.377 |  |
| rs2425639 | 20 | 43027510 | *HNF4A* | A | 0.687 | 1.05 | 0.486 | 0.474 |  |
| rs2071197 | 20 | 43030435 | *HNF4A* | A | 0.713 | 0.95 | 0.443 | 0.454 |  |
| rs2071199 | 20 | 43030866 | *HNF4A* | C | 0.151 | 1.25 | 0.221 | 0.185 |  |
| rs2071200 | 20 | 43030956 | *HNF4A* | T | 0.833 | 1.03 | 0.287 | 0.281 |  |
| rs3212180 | 20 | 43034513 | *HNF4A* | G | 0.073 | 1.31 | 0.227 | 0.183 |  |
| rs736824 | 20 | 43034660 | *HNF4A* | T | 0.115 | 1.24 | 0.287 | 0.244 |  |
| rs745975 | 20 | 43034693 | *HNF4A* | T | 0.332 | 0.86 | 0.181 | 0.205 |  |
| rs6093976 | 20 | 43035780 | *HNF4A* | T | 0.479 | 0.89 | 0.168 | 0.185 |  |
| rs8114057 | 20 | 43036452 | *HNF4A* | A | 0.982 | 1.00 | 0.460 | 0.460 |  |
| rs11574733 | 20 | 43037422 | *HNF4A* | G | 0.556 | 1.10 | 0.209 | 0.194 |  |
| rs11574736 | 20 | 43039537 | *HNF4A* | C | 0.444 | 1.12 | 0.216 | 0.197 |  |
| rs6093978 | 20 | 43041591 | *HNF4A* | T | 0.701 | 0.95 | 0.372 | 0.383 |  |
| rs3212198 | 20 | 43044362 | *HNF4A* | T | 0.996 | 1.00 | 0.347 | 0.347 |  |
| rs3212200 | 20 | 43046929 | *HNF4A* | C | 0.563 | 0.92 | 0.260 | 0.276 |  |
| MAF: minor allele frequency  ^a^*P* value of Pearson’s chi-square test for allelic model. | | | | | | | | |  |
| ^b^Odds ratio (OR) of minor allele from two-by-two allele frequency table. | | | | | | | | |  |
